# Enhanced Fenton-like Degradation of Trichloroethylene by Hydrogen Peroxide Activated with Nanoscale Zero Valent Iron Loaded on Biochar

**DOI:** 10.1038/srep43051

**Published:** 2017-02-23

**Authors:** Jingchun Yan, Linbo Qian, Weiguo Gao, Yun Chen, Da Ouyang, Mengfang Chen

**Affiliations:** 1 Key Laboratory of Soil Environment and Pollution Remediation, Institute of Soil Science, Chinese Academy of Sciences, Nanjing 210008, China; 2 University of Chinese Academy of Sciences, Beijing 100049, China

## Abstract

Composite of nanoscale Zero Valent Iron (nZVI) loaded on Biochar (BC) was prepared and characterized as hydrogen peroxide (H_2_O_2_) activator for the degradation of trichloroethylene (TCE). nZVI is homogeneously loaded on lamellarly structured BC surfaces to form nZVI/BC with specific surface area (S_BET_) of 184.91 m^2^ g^−1^, which can efficiently activate H_2_O_2_ to achieve TCE degradation efficiency of 98.9% with TOC removal of 78.2% within 30 min under the conditions of 0.10 mmol L^−1^ TCE, 1.13 g L^−1^ nZVI/BC and 1.50 mmol L^−1^ H_2_O_2_. Test results from the Electron Spin Resonance (ESR) measurement and coumarin based fluorescent probe technology indicated that ∙OH radicals were the dominant species responsible for the degradation of TCE within the nZVI/BC-H_2_O_2_ system. Activation mechanism of the redox action of Fe^2+^/Fe^3+^ generated under both aerobic and anaerobic conditions from nZVI and single electron transfer process from BC surface bound C–OH to H_2_O_2_ promoted decomposition of H_2_O_2_ into ∙OH radicals was proposed.

As one of the most commonly used chemicals in industry, chlorinated solvents such as trichloroethylene (TCE) has been frequently encountered in subsurface environments as dense non-aqueous phase liquids (DNAPLs) at many industrial sites^1^,^2^. Due to its high toxicity and adverse effects on liver and kidney, TCE has been classified as a potential human carcinogen and listed as a priority pollutant by the United States Environmental Protection Agency (U.S. EPA)^3^, and the Safe Drinking Water Act in the USA defines the maximum contaminant level of TCE at 5 μg L^−1^ for drinking water^4^. Therefore, effective remediation and complete mineralization of TCE in aquifers is urgently required to reduce its adverse effects on the environment and human health.

Advanced oxidation processes (AOPs) have been emerged as the most efficient alternative to degrade various organic pollutants for the generation of reactive radicals^5^. Among AOPs, Fenton (i.e., the reaction between Fe^2+^ and H_2_O_2_) is a powerful oxidant generating the hydroxyl radicals (∙OH, E_0_ = 2.80 V), which react with various organic compounds at the near-diffusion controlled rates^6^,^7^, leading to an effective degradation and mineralization of organic pollutants^8^. However, it should to be operated at pH < 3.0 and the generated iron sludge limited the wide application of the homogeneous Fenton process^9^. Heterogeneous Fenton-like activator of zero valent iron (ZVI) was developed, which could be used over a wide pH range to decompose H_2_O_2_ instead of homogeneous ferrous iron^10^,^11^. In addition, nanoscale zero valent iron (nZVI) could enhance H_2_O_2_ activation due to the small particle size and high reactivity. Xu *et al*.^12^ reported that the heterogeneous Fenton-like system using nZVI as catalyst was effective for the removal of biocide 4-chloro-3-methyl phenol in the presence of H_2_O_2_, and the reaction was induced through following reactions in the heterogeneous system of nZVI/H_2_O_2_:


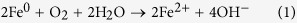



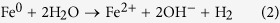



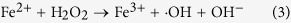


Though nZVI has performance for H_2_O_2_ activation, it tends to aggregate into forming microscale particles due to its high surface energy and strong magnetic interaction, leading to the reduced reactivity^13^. To overcome the problem, granular activated carbon^14^, bentonite^15^, and rectorite^16^ were introduced as a support for nZVI to gain better distribution. Biochar (BC) is a promising environmental friendly material pyrolyzed under low oxygen conditions. It possesses large surface area with porous structure and has oxygen containing functional groups^17^. Thus, it is anticipated that nZVI loaded uniformly on BC surface to form nZVI/BC composite will effectively prevent the aggregation of nZVI with significantly enhanced performance.

In this study, the composite of nZVI loaded on BC sheets was synthesized and characterized as H_2_O_2_ activator for the degradation of TCE. The presence of C−OH groups on BC surface could be used to activate H_2_O_2_ through electron transfer process^18^. Thus, both the dispersive nature and H_2_O_2_ activator of BC will be simultaneously achieved for nZVI/BC. The present work aims to (1) synthesize a novel composite of nZVI/BC, where nZVI was loaded on BC surface uniformly and the aggregation of nZVI was prevented effectively, (2) characterize nZVI/BC activation ability for H_2_O_2_ to degrade TCE in aqueous solution, and (3) explore the activation mechanisms of H_2_O_2_ in the presence of nZVI/BC.

## Results and Discussion

### Characterization of nZVI/BC

**Figure 1:**
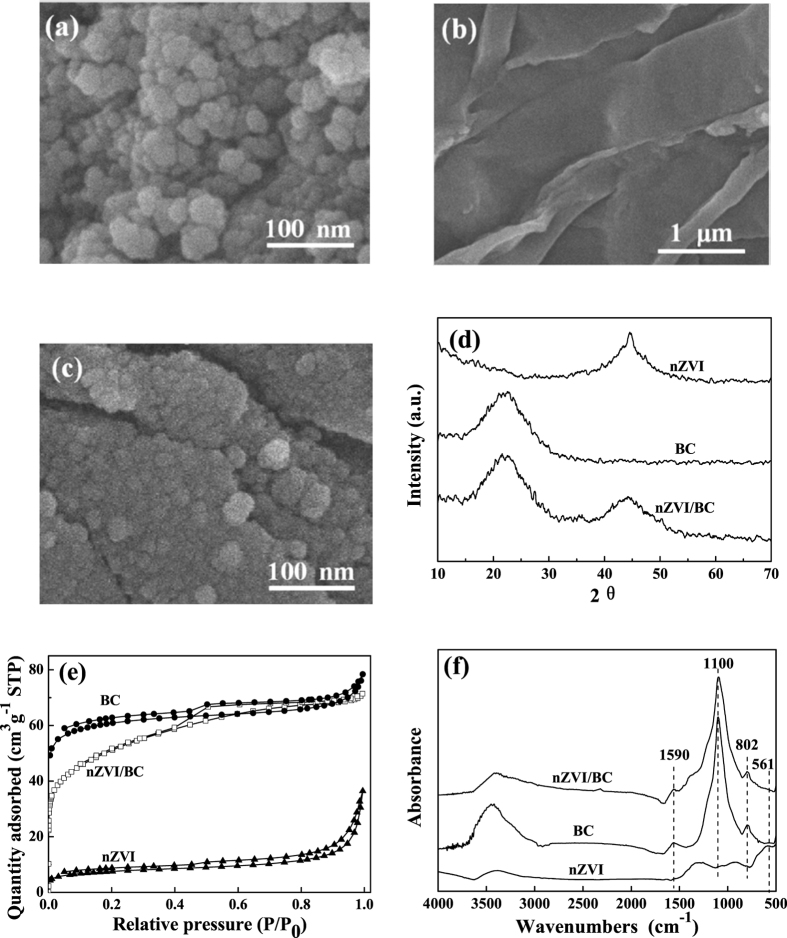
SEM images of (**a**) nZVI, (**b**) BC, (**c**) nZVI/BC, (**d**) XRD patterns of nZVI, BC and nZVI/BC, (**e**) nitrogen adsorption-desorption isotherms of nZVI, BC and nZVI/BC, and (**f**) FT-IR spectra of nZVI, BC and nZVI/BC.

SEM analyses were firstly conducted to observe the morphologies of the prepared nZVI, BC and nZVI/BC, respectively. As illustrated in Fig. 1a, nZVI was spherical with diameters of about 30 nm, and the agglomeration of nZVI was observed due to the nanometer effect and magnetic properties of nZVI. In addition, lamellarly structured BC of rough surface morphologies was obtained, and nZVI was homogeneously loaded on BC surface from the SEM image of nZVI/BC composite. XRD was also conducted, and the data were shown in Fig. 1d. It can be seen that the XRD pattern of nZVI revealed a highly crystalline and single phase structure by diffraction peaks at 45.0° (JCPD 01-087-0721)^19^. The crystallite size of nZVI was estimated to be 29.7 nm derived from the Debye-Sherrer equation (D = Kλ/(βcosθ), where K is the Sherrer constant (0.89), λ is the X-ray wavelength (0.15418 nm), β is the full peak width at half maximum and θ is the Bragg diffraction angle), which was in accordance with the SEM image. The broad reflection peak of BC in XRD indicated the amorphous BC, suggesting that nZVI was successfully loaded on BC surface from XRD spectrum of nZVI/BC (Fig. 1d).

The BET surface areas were measured by using N_2_ adsorption method as shown in Fig. 1e. The S_BET_ values were calculated according to 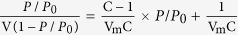, where V is the volume of nitrogen adsorbed per gram, V_m_ is the monolayer capacity and C is related to the heat of adsorption. From the results, the S_BET_ value of bare nZVI was 26.61 m^2^ g^−1^, and was increased to 184.91 m^2^ g^−1^ for the nZVI/BC composite after nZVI was loaded onto BC surface (S_BET_ value of 205.35 m^2^ g^−1^). In the FTIR spectrum, the band at about 3400 cm^−1^ was belonged to the vibration of hydroxyl groups (−OH). The signal at 1590 cm^−1^ was ascribed to C=O stretching vibration, the peak at 1100 cm^−1^ was assigned to the C−O groups, and the absorption peak at 802 cm^−1^ was corresponded to the vibration of aromatic C−H^17^. The weak adsorption in nZVI/BC spectrum at 561 cm^−1^ was observed in the Fig. 1f, indicating the Fe−O bond formed between BC and nZVI^20^.

### Heterogeneous fenton-like degradation of TCE in nZVI/BC-H_2_O_2_ system

**Figure 2:**
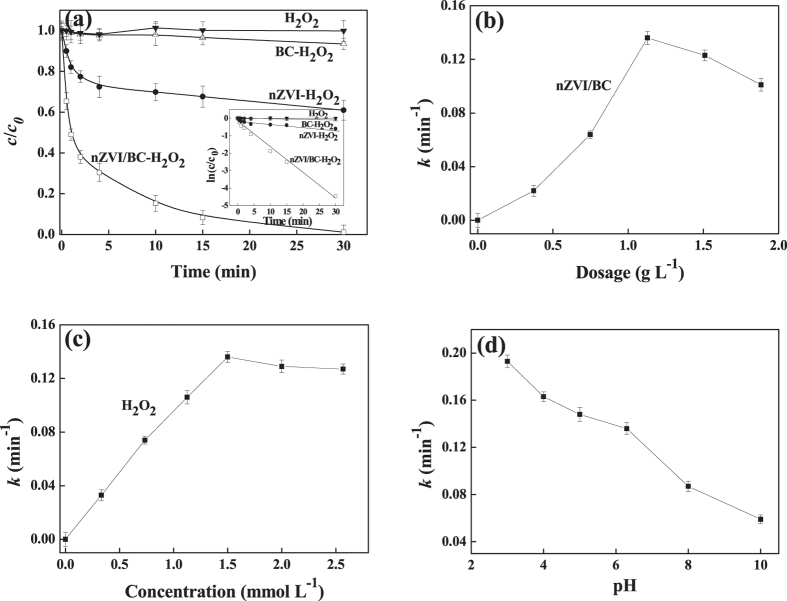
(**a**) Kinetic data of TCE in the systems of H_2_O_2_, nZVI-H_2_O_2_, BC-H_2_O_2_ and nZVI/BC-H_2_O_2_, and the inset was the plot of ln(c/c_0_) vs. t within the four mentioned systems, effects of (**b**) nZVI/BC dosage, (**c**) H_2_O_2_ concentration and (**d**) solution pH on apparent reaction rate constant of TCE degradation. Reaction conditions: the concentration of nZVI itself or in nZVI/BC composite was 0.19 g L^−1^, the dosage of nZVI/BC was 1.13 g L^−1^, the dosage of BC was 0.94 g L^−1^, the concentration of H_2_O_2_ was 1.50 mmol L^−1^, the concentration of TCE was 0.10 mmol L^−1^, and the initial pH was 6.2.

The performances of TCE degradation by H_2_O_2_ activated with nZVI, BC and nZVI/BC were investigated and presented in Fig. 2a. The control test suggested the TCE (0.10 mmol L^−1^) loss was less than 2% due to the volatilization during the experimental period under all the tested conditions. With the effect of 1.50 mmol L^−1^ H_2_O_2_, TCE was hardly degraded in the absence of any activators within 30 min, but its degradation efficiencies were enhanced to 39.1%, 6.5% and 98.9% with H_2_O_2_ in the presence of nZVI, BC and nZVI/BC, respectively. Under all the tested conditions, the TCE degradation kinetics approximately followed pseudo-first-order reaction of ln (c_0_/c_t_) = kt + b, where c_0_ and c_t_ are the TCE concentrations at initial time (t = 0) and reaction time (t = t), k is the apparent reaction rate constant (min^−1^), t is reaction time (min), and b is a constant. As illustrated in Fig. 2b by adding 0.19 g L^−1^ nZVI, the apparent reaction rate constant of TCE was 0.0128 min^−1^, and the relative small k value of TCE degradation might be due to the aggregation of prepared nZVI with a small S_BET_ value. The k value of 0.0002 min^−1^ for TCE degradation was observed when 0.94 g L^−1^ BC was added, indicating the weak activation ability of BC for H_2_O_2_, which was in accordance with the results reported by previous studies^21^,^22^. Loading nZVI onto BC significantly increased k values almost by 11 times from 0.0128 to 0.136 min^−1^ from Fig. 2a, demonstrating that nZVI/BC was more efficient than nZVI for H_2_O_2_ activation. nZVI were distributed on the BC surface homogeneously from SEM image, and nZVI/BC has higher S_BET_ value than that of the raw nZVI. The increased surface area of nZVI/BC enhanced the amount of H_2_O_2_ activation sites and TCE adsorption, which might significantly lead to the increase in the H_2_O_2_ activation ability and TCE degradation rate constant.

As shown in Fig. 2b and c, effects of nZVI/BC dosage and initial H_2_O_2_ concentration were investigated. When nZVI/BC dosage was increased from 0.372 to 1.13 g L^−1^, the k value for TCE degradation was almost linearly increased from 0.022 to 0.136 min^−1^. The increased nZVI/BC dosage provided a large amount of H_2_O_2_ active sites, and thus more ∙OH radicals generated^23^. However, the k value was decreased to 0.101 min^−1^ when the dosage of nZVI/BC was further increased to 1.13 g L^−1^. The decreased k value may be ascribed to the decrease of ∙OH radicals for TCE degradation due to the scavenging effect by excessive Fe^2+^ species (generated from Eqs 1 and 2) in according to Eq. 4^24^.


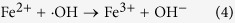



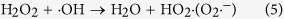


In consideration of the effect of H_2_O_2_ concentration, the k values were increased quickly from 0.033 min^−1^ to 0.136 min^−1^ as H_2_O_2_ concentrations were increased from 0.33 mmol L^−1^ to 1.50 mmol L^−1^. H_2_O_2_ is the precursor for ∙OH generation, relative high H_2_O_2_ concentration induced more ∙OH radicals accounted for TCE degradation, and hence the increased k value was obtained. When the concentration of H_2_O_2_ was beyond 1.50 mmol L^−1^, the k value was decreased due to the reaction between ∙OH and excessive H_2_O_2_ (Eq. 5)^8^,^25^. Therefore, the nZVI/BC dosage and the initial H_2_O_2_ concentration were fixed to be 1.13 g L^−1^ and 1.50 mmol L^−1^ respectively for the degradation of 0.10 mmol L^−1^ TCE.

Figure 2d showed the effect of the initial solution pH on TCE degradation in the presence of nZVI/BC and H_2_O_2_. The ability of nZVI/BC to activate H_2_O_2_ was decreased with the increase of the initial solution pH. However, the k value of 0.059 min^−1^ was observed when the solution pH was as high as 10.0, which indicated that the nZVI/BC-H_2_O_2_ system could be effective even in alkaline pH conditions with no adjustment of the pH value being needed for the effective TCE degradation.

### TCE mineralization and the stoichiometry efficiency of utilization of H_2_O_2_

**Figure 3:**
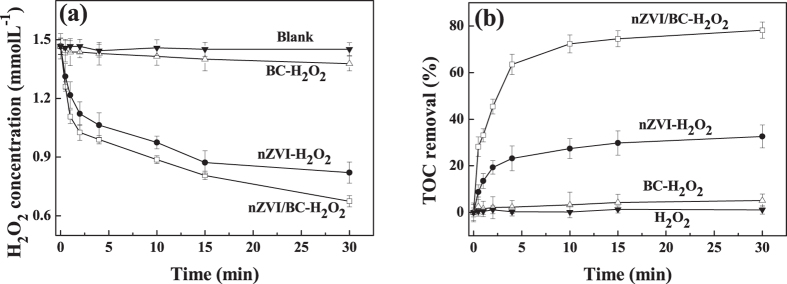
(**a**) H_2_O_2_ consumption and (**b**) TOC removal in nZVI-H_2_O_2_, BC-H_2_O_2_ and nZVI/BC-H_2_O_2_ systems. Reaction conditions: Reaction conditions: the concentration of nZVI itself or in nZVI/BC composite was 0.19 g L^−1^, the concentration of nZVI/BC was 1.13 g L^−1^, the dosage of BC was 0.94 g L^−1^, the concentration of H_2_O_2_ was 1.50 mmol L^−1^, the concentration of TCE was 0.10 mmol L^−1^, and the initial pH was 6.2.

TOC analyzer was used to evaluate the mineralization of TCE in the presence of H_2_O_2_ activated by nZVI/BC. The degradation of TCE was monitored, and the stoichiometry efficiency of utilization of H_2_O_2_ was calculated. In accordance with Eq. 6, three moles H_2_O_2_ will be consumed to obtain the complete mineralization of one mole TCE in theory. The stoichiometry efficiency of utilization of H_2_O_2_ (*η*) was the ratio of the amount of H_2_O_2_ consumed for the TCE degradation (Δ[H_2_O_2_]_degradation_) to the total amount of the H_2_O_2_ decomposed in the reaction (Δ[H_2_O_2_]_decomposition_) in accordance with Eq. 7^26^. The value of Δ[H_2_O_2_]_degradation_ was calculated by measuring the TOC change in the TCE solution. The amount of Δ[H_2_O_2_]_decomposition_ at different reaction time was measured as shown in Fig. 3a.


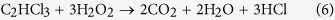



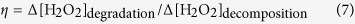


As shown in Fig. 3b in the presence of 1.50 mmol L^−1^ H_2_O_2_, the TOC removal after 30 min was 5.1%, 32.6% and 78.2% with the addition of BC, nZVI and nZVI/BC, corresponding to the TCE degradation efficiencies of 6.5%, 39.0% and 98.9% respectively from GC results (Fig. 2a), respectively. The total amount of the H_2_O_2_ decomposed in the reaction in BC-H_2_O_2_-TCE, nZVI-H_2_O_2_-TCE and nZVI/BC-H_2_O_2_-TCE systems were 1.76, 12.91 and 15.84 μmol respectively. Therefore, the calculated efficiencies for the utilization of H_2_O_2_ was 48.7%, 33.3% and 65.2% in the above three systems, respectively. The TOC removal and the efficiency for the utilization of H_2_O_2_ in nZVI/BC-H_2_O_2_-TCE system were consistently higher than those for BC-H_2_O_2_-TCE and nZVI-H_2_O_2_-TCE systems indicating the superior activation property of nZVI/BC for H_2_O_2_ after nZVI was loaded on BC surface.

### Determination of free radicals

Based on previous studies^27^,^28^,^29^,^30^, reactive oxygen species (ROSs, such as ∙OH radicals and O_2_^−^∙/HO_2_∙ radicals) were generated from the decomposition of H_2_O_2_ in homogeneous Fenton reactions at acidic and neutral conditions. In addition, high valent iron (Fe^IV^ = O) was also proposed in heterogeneous Fenton-like reaction during the activation of H_2_O_2_ by zero-valent iron under alkaline conditions^31^,^32^. To ascertain the H_2_O_2_ activation mechanism, the involved ROSs in nZVI-H_2_O_2_, BC-H_2_O_2_ and nZVI/BC-H_2_O_2_ systems were monitored by using both DMPO spin trap ESR measurement and coumarin based fluorescent probe technology.

**Figure 4:**
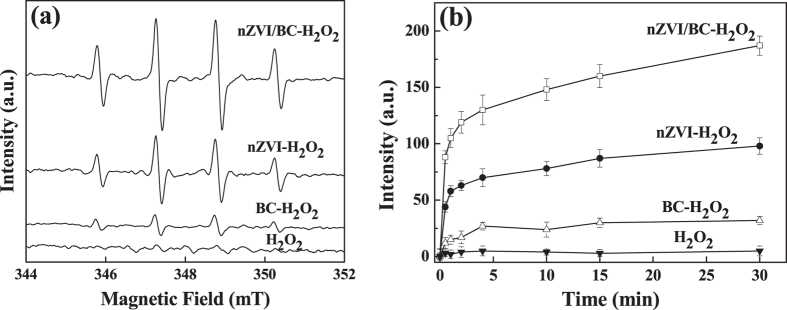
(**a**) DMPO spin-trapping ESR spectra of ∙OH radicals and (**b**) emission spectra intensity of coumarin adduct (excited at 346 nm, and detected at 456 nm) in the systems of H_2_O_2_, nZVI-H_2_O_2_, BC-H_2_O_2_ and nZVI/BC-H_2_O_2_. Reaction conditions: the concentration of nZVI itself or in nZVI/BC composite was 0.19 g L^−1^, the concentration of nZVI/BC was 1.13 g L^−1^, the dosage of BC was 0.94 g L^−1^, the concentration of H_2_O_2_ was 1.50 mmol L^−1^, the concentration of TCE was 0.10 mmol L^−1^, and the initial pH was 6.2.

As shown in Fig. 4a, no signals were observed in the presence of H_2_O_2_. The measured ESR spectra in the three systems mentioned above illustrated the four-fold characteristic peak with an intensity ratio of 1:2:2:1, which were in accordance with the pattern of typical DMPO-∙OH adduct^33^. Possibly due to the weak activation ability of BC and high adsorption of DMPO-∙OH adduct by BC in BC-H_2_O_2_ system^17^, relative low concentration of DMPO-∙OH adduct was detected in aqueous solution.

The intensity of DMPO-∙OH adduct in the nZVI/BC-H_2_O_2_ system was much higher than that in the nZVI-H_2_O_2_ system, indicating more ∙OH radicals being generated. In addition, due to the instability of the O_2_∙^−^/HO_2_∙ radicals in the solution, six-fold characteristic peak of the O_2_∙^−^/HO_2_∙ radicals adduct by using dimethyl sulfoxide as solvent was measured^34^, with no signal being detected (data were not shown). These results suggested that ∙OH radicals were the main ROSs generated from the decomposition of H_2_O_2_ responsible for TCE degradation. Coumarin based fluorescent probe technology was used to measure the generation of ∙OH radicals. As illustrated in Fig. 4b the fluorescence intensities were increased quickly in the first few minutes in both nZVI/BC-H_2_O_2_ and nZVI-H_2_O_2_ systems, indicating the fast generation of ∙OH radicals. The fluorescence intensity in the nZVI/BC-H_2_O_2_ system was consistently higher than that in the nZVI-H_2_O_2_ system, hinting the excellent property of nZVI/BC for H_2_O_2_ activation. The data obtained here was well coincided with ESR results.

### Discussion on reaction mechanism

**Figure 5:**
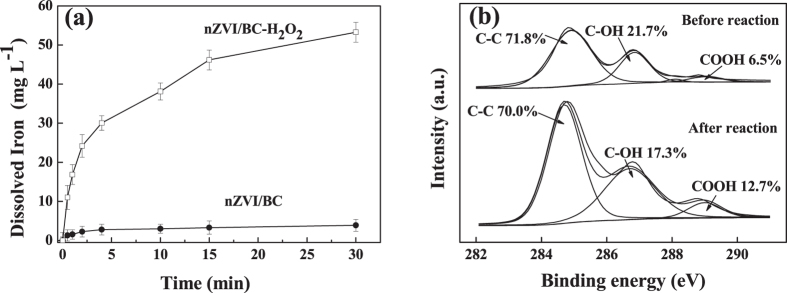
(**a**) Dissolved iron concentrations in the aqueous solution with and without H_2_O_2_ and (**b**) C 1 s XPS spectra of nZVI/BC before and after the reaction. Reaction conditions: the concentration of nZVI itself or in nZVI/BC composite was 0.19 g L^−1^, the concentration of nZVI/BC was 1.13 g L^−1^, the dosage of BC was 0.94 g L^−1^, the concentration of H_2_O_2_ was 1.50 mmol L^−1^, the concentration of TCE was 0.10 mmol L^−1^, and the initial pH was 6.2.

nZVI particles could be oxidized to Fe^2+^ under anaerobic or aerobic conditions, and it is known that homogeneous Fe^2+^ plays a critical role in the activation of H_2_O_2_ to generate ∙OH radicals^35^,^36^. Thus, the dissolved Fe^2+^ and Fe^3+^ concentrations in the presence of nZVI/BC with and without H_2_O_2_ were measured after reaction, respectively. As shown in Fig. 5a, the concentrations of Fe^2+^ and Fe^3+^ were 0.73 and 3.15 mg L^−1^ respectively with only the nZVI/BC in aqueous solution. However, in the nZVI/BC-H_2_O_2_ system, the concentrations of Fe^2+^ and Fe^3+^ were 7.70 and 45.59 mg L^−1^ respectively, being much higher than those in the absence of H_2_O_2_. The results indicated that the oxidation of nZVI to Fe^2+^ was occurred initially, and the dissolved Fe^2+^ directly activated H_2_O_2_ to generate ∙OH radicals subsequently. The consumption of Fe^2+^ for H_2_O_2_ activation accelerated nZVI transformation and Fe^3+^ formation in accordance with Eq. 3.

In the BC-H_2_O_2_ system, the degradation efficiency of TCE was 6.5%, indicating that BC has also acted as an electron-transfer mediator to activate H_2_O_2_. BC characteristics of porosity, specific surface area, surface inertness and surface functional groups might significantly affect the catalytic activity for H_2_O_2_ decomposition^37^,^38^. XPS spectra of nZVI/BC were measured to better understand the roles of BC in the activation of H_2_O_2_ before and after the reaction. As illustrated in Fig. 5b, the peaks of C (1 s) at 284.5, 286.5 and 289.0 eV were attributed to C–C, C–OH and COOH, respectively. From the spectra, the proportion of C–C, C–OH and COOH peaks were 71.8%, 21.7% and 6.5% respectively for fresh nZVI/BC before the reaction. However, the proportion of the three peaks mentioned above were 70.0%, 17.3% and 12.7% after the H_2_O_2_ activation. The decrease in surface bound C–OH proportion of BC after the reaction suggested that BC might act as an electron transfer medium participated in the H_2_O_2_ activation^39^,^40^, and the increase in COOH was due to the reaction between C–OH and H_2_O_2_ in accordance with Eq. 8^18^. By releasing organic radicals of CO∙ from C–OH through single electron transfer process, ∙OH radicals were generated (Eq. 9)^41^.


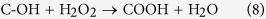



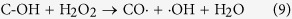


**Figure 6:**
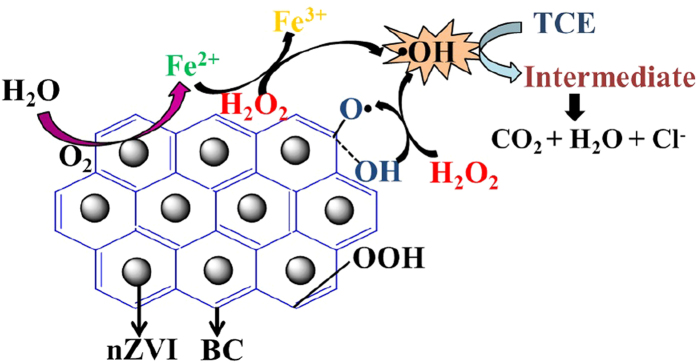
A proposed mechanism for TCE degradation by the nZVI/BC activation of H_2_O_2_.

As derived from the above discussions, the activation mechanism of H_2_O_2_ in the presence of nZVI/BC was proposed in Fig. 6. Firstly, nZVI particles were oxidized to Fe^2+^, and the redox reaction of Fe^2+^/Fe^3+^ was accounted for the generation of ∙OH radicals. Secondly, as an electron transfer mediator to H_2_O_2_, BC surface bound C–OH decomposed H_2_O_2_ into ∙OH radicals by releasing CO∙ radicals. Once ∙OH radicals having been produced, it would rapidly react with TCE. GC-MS was utilized to monitor the process of TCE degradation, however, no intermediate products was detected except for the undegraded TCE. Though TCE would be transformed into low molecule weight organic acids with the effect of ∙OH radicals initially, only CO_2_ and Cl^−^ were measured during the oxidative process in the nZVI/BC-H_2_O_2_-TCE system.

## Conclusions

The nZVI/BC composite was successfully synthesized and characterized as an efficient H_2_O_2_ activator for the degradation of TCE. nZVI loaded on lamellarly structured BC surface prevented its agglomeration behaviour, whcih significantly enhanced the generation of ∙OH radicals. The redox effect of Fe^2+^/Fe^3+^ and the single electron transfer process from BC surface bound C–OH to H_2_O_2_ were accounted for the promoted generation of ∙OH radicals, leading to rapid TCE degradation. The enhanced Fenton-like activation of H_2_O_2_ using nZVI/BC presents the great potential for TCE degradation in aqueous solution.

## Materials and Methods

### Chemicals and materials

FeSO_4_ ∙ 7H_2_O, NaBH_4_, H_2_O_2_ (30%, w/w), 5,5-Dimethyl-1-pyrrolidine N-oxide (DMPO) and trichloroethylene (TCE, 99.0%) were purchased from Sinopharm Chemical Reagent Co. Ltd. HCl and NaOH were obtained from Shanghai Lingfeng Chemical Reagent Co. Ltd. All chemicals used in this work were used as received without further purification.

### Synthesis of nZVI/BC composite

Biochars were prepared by the pyrolysis of rice hull collected locally. Firstly, the rice hull was washed with ultrapure water and dried in oven at 80 °C. Secondly, the dried rice hull was pyrolyzed in muffle furnace under oxygen limited condition at a temperature of 350 °C for 6 h. Finally, the BC were obtained after treating with 1.0 mol L^−1^ HCl and washed with ultrapure water.

**Figure 7:**
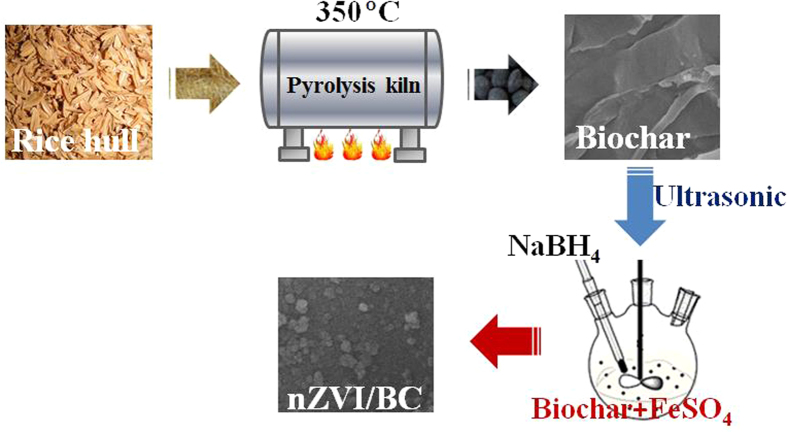
The schematic for the preparation of nZVI/BC composite.

For the synthesis of nZVI/BC composite, 3.78 g biochar was dispersed in 250 mL oxygen free ultrapure water. Then, 0.0135 mol FeSO_4_ ∙ 7H_2_O was added at pH 5.0. With mechanical stirring and ultrasonic, nZVI was formed and loaded on the surface of BC by addition of 100 mL 0.27 mol L^−1^ NaBH_4_ at a velocity of 20 mL min^−1^. Following 2 h reaction, the nZVI/BC composite were separated and washed with deoxygenized ultrapure water, and finally vacuum dried. The preparation of nZVI was described as Eq. 10, and the schematic for the preparation of nZVI/BC composite was shown in Fig. 7.


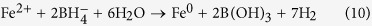


### Characterization

X-ray diffraction (XRD, X’TRA, Swiss) analysis was conducted to determine the crystal structure and crystallinity of the prepared composites using Cu K_α_ radiation with 2θ collection range from 10° to 80°. X-ray photoelectron spectra (XPS) were recorded on Axis Ultra spectrometer (Kratos) using Al Kα radiation excitation source. The infrared spectrum was recorded on Fourier transform infrared spectroscopy (FT-IR) spectra from 400 to 4000 cm^−1^ (NICOLET iN10 MX, Thermo Scientific, USA). The morphology of the composites were observed using scanning electron microscope (SEM, Hitachi S-4800, Japan) with 10 kV accelerating voltage, and the Brunauer-Emmett-Teller (BET) specific surface areas (S_BET_) were measured with ASAP 2020M + C (Micromeritics, USA) from N_2_ adsorption method.

### Procedures and analysis

In a typical sacrificial batch experiment, a 20 mL cylindrical glass vessel was fully filled with 0.10 mmol L^−1^ TCE, appropriate amounts of H_2_O_2_ and activators (i.e., nZVI, BC or nZVI/BC), and the vessel was tightly sealed with Teflon reaction head successively. Then, the reaction was initiated in a rotary shaker at 298 K and 150 rpm. Control test was also carried out under the same condition without H_2_O_2_ and activators. Samples were taken at the desired reaction time intervals and filtered through 0.2 μm membrane prior to the analysis. Samples were conducted in triplicate and the mean value was obtained.

The concentration of TCE was analyzed by headspace Gas Chromatograph Mass Spectrometer (GC-MS, Agilent 7890A and 5975C) using DB-624 chromatographic column (30.0 m × 0.25 mm × 1.4 μm). Dissolved ferrous ion was quantified through 1,10-Phenanthrolinemonohydrate Spectrophotometry by using a Cary 50 UV-vis spectrophotometer (Varian Cary 50, USA). The concentration of H_2_O_2_ was measured with the DPD method using UV-vis spectrophotometer (Varian Cary 50, USA). The generated radical species was detected with electron spin resonance spectrometer (Bruker ESR 300E, Germany) with microwave bridge (receiver gain, 1 × 10^5^; modulation amplitude, 2 Gauss; microwave power, 10 mW; modulation frequency, 100 kHz) using DMPO as radical spin-trapping reagent, and fluorescence spectra were measured on fluorescence spectrophotometer (Jasco FP-6200, Japan). Total organic carbon (TOC) was recorded with a multi N/C model TOC analyzer (Analytik Jena, multi N/C 2100, Germany).

## Additional Information

**How to cite this article**: Yan, J. *et al*. Enhanced Fenton-like Degradation of Trichloroethylene by Hydrogen Peroxide Activated with Nanoscale Zero Valent Iron Loaded on Biochar. *Sci. Rep.* **7**, 43051; doi: 10.1038/srep43051 (2017).

**Publisher's note:** Springer Nature remains neutral with regard to jurisdictional claims in published maps and institutional affiliations.

## References

[b1] Lu R. . Determination of chlorinated hydrocarbons in water using highly sensitive mid-infrared sensor technology. Scientific Reports 3, 2525 (2013). 23982222 10.1038/srep02525 PMC3755290

[b2] Yan J. C. . Degradation of trichloroethylene by activated persulfate using a reduced graphene oxide supported magnetite nanoparticle. Chem. Eng. J. 295, 309–316 (2016).

[b3] Lee Y. & Lee W. Degradation of trichloroethylene by Fe(II) chelated with cross-linked chitosan in a modified Fenton reaction. J. Hazard. Mater. 178, 187–193 (2010). 20129729 10.1016/j.jhazmat.2010.01.062

[b4] US Environmental Protection Agency. *Edition of the Drinking Water Standards and Health Advisories. EPA 822-R-09-011*. EPA Office of Water: Washington DC (2009).

[b5] Yan J. C. . Degradation of sulfamonomethoxine with Fe_3_O_4_ magnetic nanoparticles as heterogeneous activator of persulfate. J. Hazard. Mater. 186, 1398–1404 (2011). 21237557 10.1016/j.jhazmat.2010.12.017

[b6] Andreozzi R. . Advanced oxidation processes (AOP) for water purification and recovery. Catal. Today 53, 51–59 (1999).

[b7] Pestovsky O.& Bakac A. Aqueous ferryl(IV) ion: kinetics of oxygen atom transfer to substrates and oxo exchange with solvent water. Inorg. Chem. 45, 814–820 (2006). 16411719 10.1021/ic051868z

[b8] Yan J. C. . Efficient degradation of organic pollutants with ferrous hydroxide colloids as heterogeneous Fenton-like activator of hydrogen peroxide. Chemosphere 87, 111–117 (2012). 22192793 10.1016/j.chemosphere.2011.11.069

[b9] Nie Y. . Enhanced Fenton-like degradation of refractory organic compounds by surface complex formation of LaFeO_3_ and H_2_O_2_. J. Hazard. Mater. 294, 195–200 (2015). 25867592 10.1016/j.jhazmat.2015.03.065

[b10] Bremner D. H. . Phenol degradation using hydroxyl radicals generated from zero-valent iron and hydrogen peroxide. Appl. Catal. B-Environ. 63, 15–19 (2006).

[b11] Segura Y., Martínez F. & Melero J. A. Effective pharmaceutical wastewater degradation by Fenton oxidation with zero-valent iron. Appl. Catal. B-Environ. 136–137, 64–69 (2013).

[b12] Xu L. J. & Wang J. L. A heterogeneous Fenton-like system with nanoparticulate zero-valent iron for removal of 4-chloro-3-methyl phenol. J. Hazard. Mater. 186, 256–264 (2011). 21109349 10.1016/j.jhazmat.2010.10.116

[b13] Fu F., Dionysiou D. D. & Liu H. The use of zero-valent iron for groundwater remediation and wastewater treatment: a review, J. Hazard. Mater. 267, 194–205 (2014). 24457611 10.1016/j.jhazmat.2013.12.062

[b14] Mackenzie K. . Carbo-Iron - An Fe/AC composite - As alternative to nano-iron for groundwater treatment. Water Res. 46, 3817–3826 (2012). 22591820 10.1016/j.watres.2012.04.013

[b15] González-Bahamón L. F. . New Fe-immobilized natural bentonite plate used as photo-Fenton catalyst for organic pollutant degradation. Chemosphere 82, 1185–1189 (2011). 21167551 10.1016/j.chemosphere.2010.11.071

[b16] Luo S. . Synthesis of reactive nanoscale zero valent iron using rectorite supports and its application for Orange II removal. Chem. Eng. J. 223, 1–7 (2013).

[b17] Yan J. C. . Biochar supported nanoscale zerovalent iron composite used as persulfate activator for removing trichloroethylene. Bioresour. Technol. 175, 269–274 (2015). 25459832 10.1016/j.biortech.2014.10.103

[b18] Fang G. D. . New Insights into the mechanism of the catalytic decomposition of hydrogen peroxide by activated carbon: implications for degradation of diethyl phthalate. Ind. Eng. Chem. Res. 53, 19925–19933 (2014).

[b19] Hoch L. B. . Carbothermal synthesis of carbon-supported nanoscale zero-valent iron particles for the remediation of hexavalent chromium. Environ. Sci. Technol. 42, 2600–2605 (2008). 18505003 10.1021/es702589u

[b20] Liu Z. G., Zhang F. S. & Wu J. Z. Characterization and application of chars produced from pinewood pyrolysis and hydrothermal treatment. Fuel 89, 510–514 (2010).

[b21] Kan E. & Huling S. G. Effects of temperature and acidic pretreatment on Fenton-driven oxidation of MTBE-spent Granular activated carbon. Environ. Sci. Technol. 43, 1493–1499 (2009). 19350925 10.1021/es802360f

[b22] Duarte F., Maldonado-Hódar F. J. & Madeira L. M. New insight about orange II elimination by characterization of spent activated carbon/Fe Fenton-like catalysts. Appl. Catal. B-Environ. 129, 264–272 (2013).

[b23] Moroto J. M. R. . Kinetics of the chemical reduction of nitrate by zero valent iron. Chemosphere 74, 804–809 (2009). 19041116 10.1016/j.chemosphere.2008.10.020

[b24] Zhou T. . Oxidation of 4-chlorophenol in a heterogeneous zero valent iron/H_2_O_2_ Fenton system: kinetic, pathway and effect factors. Sep. Purif. Technol. 62, 551–558 (2008).

[b25] Xu L. J. & Wang J. L. Magnetic nanoscaled Fe_3_O_4_/CeO_2_ composite as an efficient Fenton-like heterogeneous catalyst for degradation of 4-chlorophenol. Environ. Sci. Technol. 46, 10145–10153 (2012). 22924545 10.1021/es300303f

[b26] Zhang X. Y. . Degradation of bisphenol A by hydrogen peroxide activated with CuFeO_2_ microparticles as a heterogeneous Fenton-like catalyst: Efficiency, stability and mechanism. Chem. Eng. J. 236, 251–262 (2014).

[b27] Haber F. & Weiss J. The catalytic decomposition of hydrogen peroxide by ferrous salts. Proc. R. Soc. Lond. Ser. A. 147, 332–351 (1934).

[b28] Zhang X. . Degradation of trichloroethylene in aqueous solution by calcium peroxide activated with ferrous ion. J. Hazard. Mater. 284, 253–260 (2015). 25463240 10.1016/j.jhazmat.2014.11.030

[b29] Duesterberg C. K. & Waite T. D. Process optimization of Fenton oxidation using kinetic modeling. Environ. Sci. Technol. 40, 4189–4195 (2006). 16856734 10.1021/es060311v

[b30] Song K. . Role of oxidants in enhancing dewaterability of anaerobically digested sludge through Fe (II) activated oxidation processes: hydrogen peroxide versus persulfate. Scientific Reports 6, 24800 (2016). 27109500 10.1038/srep24800 PMC4842980

[b31] Keenan C. R. & Sedlak D. L. Factors affecting the yields of oxidants from the reaction of nanoparticulate zero-valent iron and oxygen. Environ. Sci. Technol. 42, 1262–1267 (2008). 18351103 10.1021/es7025664

[b32] Keenan C. R. & Sedlak D. L. Ligand-enhanced reactive oxidant generation by nanoparticulate zero-valent iron and oxygen. Environ. Sci. Technol. 42, 6936–6941 (2008). 18853812 10.1021/es801438f PMC2701397

[b33] Ma W. . Efficient degradation of organic pollutants by using dioxygen activated by resin-exchanged iron(II) bipyridine under visible irradiation. Angew. Chem. Int. Ed. 115, 1059–1062 (2003). 10.1002/anie.200390264 12616558

[b34] Chen C. . Effect of transition metal ions on the TiO_2_-assisted photodegradation of dyes under visible irradiation: A probe for the interfacial electron transfer process and reaction mechanism. J. Phys. Chem. B 106, 318–324 (2001).

[b35] Kim J. Y. . Inactivation of MS2 coliphage by Fenton’s reagent. Water Res. 44, 2647–2653 (2010). 20172583 10.1016/j.watres.2010.01.025

[b36] Nieto-Juarez J. I. . Inactivation of MS2 coliphage in Fenton and Fenton-like systems: role of transition metals, hydrogen peroxide and sunlight. Environ. Sci. Technol. 44, 3351–3356 (2010). 20356037 10.1021/es903739f

[b37] Rey A. . Influence of the structural and surface characteristics of activated carbon on the catalytic decomposition of hydrogen peroxide. Appl. Catal. A: Gen. 402, 146–155 (2011).

[b38] Ribeiro R. S. . The influence of structure and surface chemistry of carbon materials on the decomposition of hydrogen peroxide. Carbon 62, 97–108 (2013).

[b39] Domínguez C. M. . Highly efficient application of activated carbon as catalyst for wet peroxide oxidation. Appl. Catal. B-Environ. 140–141, 663–670 (2013).

[b40] Domínguez C. M. . The use of cyclic voltammetry to assess the activity of carbon materials for hydrogen peroxide decomposition. Carbon 60, 76–83 (2013).

[b41] Fang G. D. . Key role of persistent free radicals in hydrogen peroxide activation by biochar: Implications to organic contaminant degradation. Environ. Sci. Technol. 48, 1902–1910 (2014). 24422431 10.1021/es4048126

